# Changes in Documentation After Implementing Open Notes in Mental Health Care: Pre-Post Mixed Methods Study

**DOI:** 10.2196/72667

**Published:** 2025-09-03

**Authors:** Eva Meier-Diedrich, Charlotte Blease, Martin Heinze, Jonas Wördemann, Julian Schwarz

**Affiliations:** 1 Department of Psychiatry and Psychotherapy, Center for Mental Health Immanuel Hospital Rüdersdorf Brandenburg Medical School Theodor Fontane Rüdersdorf Germany; 2 Faculty of Health Sciences Brandenburg Brandenburg Medical School Theodor Fontane Rüdersdorf Germany; 3 Participatory eHealth and Health Data Research Group Department of Women's and Children's Health Uppsala University Uppsala Sweden; 4 Center for Health Service Research Brandenburg Brandenburg Medical School Theodor Fontane Rüdersdorf Germany

**Keywords:** clinical documentation, open record access, electronic health record, psychotherapy, psychiatry, health care, patient accessible health record, eHealth, patient portal

## Abstract

**Background:**

The practice of providing patients with digital access to clinical narrative documentation by health care professionals (HCPs) is known as open notes. In mental health care, this innovation has the potential to increase transparency and foster greater trust in the treatment process. While open notes may improve the quality of care and patient engagement, some HCPs are concerned that they may change the nature of clinical documentation and compromise its quality.

**Objective:**

This study aims to examine potential objective and subjective changes in clinical documentation following the implementation of open notes.

**Methods:**

Clinical notes written before and after the implementation of a patient portal with open notes function in 3 psychiatric outpatient clinics in Germany were collected. A total of 876 notes (453 prenotes and 423 postnotes) were rated on 16 linguistic features using a Likert scale. Differences were analyzed using the Wilcoxon signed rank test. In addition, 10 in-depth qualitative interviews with psychiatric HCPs were conducted and analyzed using reflexive thematic analysis.

**Results:**

Postimplementation significant differences were found in several linguistic features: Monoglossic (*P*=.002), incomprehensible (*P*<.001), demeaning (*P*<.001), stigmatizing (*P*<.001), factual (*P*<.001), and controlling (*P*=.002) language decreased, while comprehensible (*P*<.001), resource-oriented (*P*<.001), heteroglossic (*P*<.001), personal (*P*<.001), and emotional positive (*P*=.047) language increased. Interviewed HCPs reported noticeable changes in both their clinical notes and documentation practices. They described reducing the use of medical jargon, providing more detailed explanations, and tailoring documentation to better meet patient needs, resulting in slightly longer notes. However, in the subjective perception of the HCPs, the information they documented in the clinical notes remained mostly the same. HCPs noted an increase in time and workload associated with the new documentation approach, partly due to the workflow adjustments required to adapt to open notes.

**Conclusions:**

To our knowledge, this is the first study to systematically analyze quantitative documentation changes in the field of mental health. The implementation of open notes seems to result in both objective and subjective changes in clinical documentation and documentation practices. Quantitative and qualitative findings from our study suggest that HCPs generally strove to create more patient-friendly notes. In practice, this may benefit both patients and the therapeutic relationship. For open notes to be sustainable in practice, they must be seamlessly and efficiently integrated into HCPs' daily workflows. This requires not only structural changes, but also educating HCPs—both during their training and in clinical practice—on how to write open notes in a way that is both effective and patient-friendly.

**Trial Registration:**

German Register of Clinical Studies DRKS00030188; https://tinyurl.com/mum4djbe

## Introduction

As the digital transformation of health care progresses, an increasing number of countries are providing patients with access to their electronic health records [1]. This web-based record access can extend beyond viewing basic health information, such as lab results and medication plans, to include functionalities—such as the ability to access clinical notes written by health care professionals (HCPs). The practice of patients reading their HCPs’ documentation through secure web-based patient portals is known as open notes [2]. Open notes are already standard practice in the United States and several Scandinavian countries [3]. In the United Kingdom, patients have had access to their general practitioners’ clinical notes since 2022 through the National Health Service App [4], while in Switzerland, inpatient psychiatric clinics are now mandated to offer open notes to their patients [5].

Existing research indicates that open notes can positively affect patient care by improving patient activation [6], engagement [7], empowerment [8], and medication adherence [9]. Furthermore, open notes have been linked to strengthening the therapeutic relationship [5]. The domain of psychiatry and mental health care represents a particularly compelling context for the adoption of open notes. Patients in these settings often face societal stigma and, in some cases, coercion during treatment [10,11]. Thus, they may derive considerable benefit from increased transparency and involvement in their care. Studies suggest that open notes can foster trust in HCPs, enhance health literacy, and promote greater empowerment among psychiatric patients [12]. Moreover, open notes have been found to improve patients’ understanding of their mental health conditions and the treatments they are undergoing [13]. However, some studies also show that patients dislike finding surprising, nondisclosed information or notes that are disrespectful [14].

While many patients support the implementation of open notes, HCPs—especially those in psychiatric settings—express mixed attitudes toward their adoption [5,12]. Some HCPs approach open notes with curiosity and a willingness to engage, while others remain skeptical and concerned [15]. Common concerns include increased workload, driven by the need to adapt documentation into more patient-friendly language, avoid jargon, and provide clearer explanations [15,16]. Additionally, HCPs worry that open notes may compromise documentation quality, leading them to write less candidly or omit sensitive information to avoid distressing patients [17,18]. Conversely, some HCPs view open notes as an opportunity to develop more patient-centered documentation and promote greater care transparency [19].

A recent scoping review highlights that open notes can lead to changes in documentation length, comprehensibility, accuracy, tone, and wording, as well as its candor, quality, and utility as a professional tool [20]. However, the direction of these changes remains inconclusive [21]. Despite growing interest in open notes, several important gaps in the literature remain. First, many studies focus on anticipated effects rather than actual experiences of open notes in practice [22]. Second, the perspectives of (psychiatric) HCPs are often underrepresented, with much of the research emphasizing patient experiences [5,23]. Third, the majority of studies rely on self-reported perceptions rather than on analyzing actual documentation and clinical notes [22]. Moreover, research that examines changes in clinical documentation within psychiatric settings remains limited and often yields inconclusive results [24-26]. To our knowledge, no prior study in the mental health field has conducted a quantitative document analysis of open notes and examined objective changes in documentation.

Building on this existing body of research, the present study explores two research questions: (1) How does clinical documentation change in content and language after the introduction of Open Notes? (2) How do psychiatrists and psychologists adapt their documentation practices after implementing Open Notes?

## Methods

### Design

This study is part of the PEPPPSY project (June 2021 to December 2026), which focuses on “Piloting and Evaluating a Participatory Patient Record in Psychiatry and Somatic Medicine” [27,28]. The primary aim of PEPPPSY is to investigate the development, implementation, processes, and outcomes of a digital patient portal from the perspectives of patients and HCPs. Given the exploratory nature of this study branch, a mixed methods approach was used to comprehensively analyze changes in clinical documentation following the implementation of open notes. This study uses a convergent parallel design. This involved collecting and analyzing qualitative and quantitative data independently but concurrently. The data were then compared and interpreted together to gain a more comprehensive understanding of the observed changes [29].

### Ethical Considerations

Ethical approval for this study was obtained from the Ethics Committee of the Medical University of Brandenburg (reference number E-01-20210727), and the study was registered with the German Clinical Trial Register (DRKS00030188).

### Study Setting

The study was conducted at 3 outpatient university clinics of Psychiatry and Psychotherapy at Brandenburg Medical School, Immanuel Hospital Rüdersdorf, located in the rural Berlin/Brandenburg area. These clinics serve a population of approximately 255,000 residents in the surrounding region. These clinics specialize in comprehensive mental health services, addressing complex cases experiencing chronic or severe mental health conditions requiring multidisciplinary care.

### PEPPPSY App

The pilot patient portal PEPPPSY is a digital test environment specially developed at the Brandenburg Medical School to test innovative forms of patient health data access. It is a standalone web-based platform designed to provide research-focused access to selected components of patients’ digital health records. PEPPPSY was created through an iterative process involving participatory design, development, application, and evaluation [27]. Its core feature allows patients to securely view their clinical notes. In this study, HCPs completed standard documentation in the internal clinical system and could transfer notes to the PEPPPSY portal either by copying and pasting or by tailoring the content for patient readability. Access to the portal required 2-factor authentication.

### Recruitment and Eligibility

HCPs at the study centers were directly approached by the study team and provided with information about the study’s objectives, procedures, and compensation (€50 [US $52.04] for general participation in the PEPPPSY study and an additional €50 [US $52.04] for taking part in an interview). Those who agreed to participate gave informed consent and received an introduction to the patient portal from a study team member. Typically, HCPs accessed the patient portal using their professional computers or mobile devices. In order to qualify for participation, HCPs were required to be employed at one of the study centers. To be considered for the interview study, participating HCPs were required to have written and published at least one open note.

Patients were recruited through purposive sampling, selecting individuals who met the following criteria: at least 18 years of age, diagnosis of a severe mental illness according to the German version of the *ICD-10* (*International Statistical Classification of Diseases, Tenth Revision*) criteria, ongoing outpatient treatment at one of the study centers within the past 6 months and ability to provide informed consent. Eligibility assessments were conducted by qualified participating HCPs (psychiatrists and psychotherapists). Patients presenting with suicidal tendencies, posing a risk to others, or experiencing psychosis or severe cognitive impairments—regardless of their specific diagnosis—were excluded. Eligible patients were approached in person by their HCPs and received detailed information about the study’s objectives and procedures. Those willing to participate provided written informed consent. After enrollment, patients were introduced to the patient portal either by their HCP or a study team member and were granted access. They also received a written user manual containing step-by-step guidance and screenshots. The study team remained available to assist both patients and HCPs with technical or procedural questions. Most patients accessed the portal using their personal digital devices (eg, smartphones or laptops). For those without personal devices, a study tablet was available at the study center. However, all participants were required to have a device capable of receiving text messages, as secure 2-factor authentication was necessary for logging into the patient portal.

### Data Collection

For the comparison of “closed” clinical notes (prenotes) and open notes (postnotes), clinical notes for each participating patient were retrieved from the internal clinical documentation system for prenotes (November 2022-December 2023) and from the PEPPPSY portal for postnotes (January 2023-February 2024). Participating patients completed sociodemographic questionnaires and pre and postintervention surveys; these results will be reported elsewhere.

In addition, qualitative interviews were conducted with HCPs by EM using a semistructured interview guide to gain deeper insights into potential changes in documentation. Interviews lasted 20-30 minutes, were recorded, transcribed, and anonymized. Additional sociodemographic data were collected from HCPs.

### Data Analysis

For the document analysis (comparing pre- and postnotes matched at the patient level), pre and postnotes were rated by 2 independent raters on 16 linguistic characteristics using a 3-point Likert scale (1=not present, 2=neutral, 3=present). The rating always reflected the overall manifestation of each linguistic characteristic within a given note. The items were derived from recent research on open notes and linguistic analyses and were organized into contrasting pairs: comprehensible versus incomprehensible language, appreciative versus demeaning language, resource-oriented versus deficit-oriented language, positive versus negative emotional language, empowering versus controlling language, stigmatizing versus destigmatizing language, and personal versus factual language, monoglossic versus heteroglossic language (monoglossic language refers to communication that presents a single, authoritative perspective, often stating facts or opinions as absolute). Heteroglossic language, on the other hand, includes multiple perspectives, voices, or levels of uncertainty, acknowledging different viewpoints or possibilities. Multimedia Appendix 1 [17,21,23,24,30-93] provides detailed definitions of the linguistic characteristics and the scientific sources used to develop them. The selected dichotomies are based on recent research on open notes and linguistic analysis, capturing key linguistic contrasts relevant to patient communication. Organizing the items into contrasting pairs enables a structured analysis and provides practical insights for improving communication in open notes.

To ensure consistency in applying the coding framework, both raters participated in extensive joint training and calibration sessions using a shared set of sample notes. Following the procedure recommended by O’Connor and Joffe [94], the raters initially double-coded a small portion of the data to identify and resolve potential issues in the coding scheme. Discrepancies were discussed collaboratively, and the coding manual was iteratively refined to enhance clarity and alignment. Then, both raters independently coded a random 10% subset of clinical notes, yielding a Cohen κ of 0.80. According to Landis and Koch [95], this indicates strong interrater agreement. Based on this strong level of agreement, the remaining data were divided, with each rater independently coding half of the clinical notes.

The normal distribution for each item was visually assessed using histograms. A Wilcoxon signed rank test was performed using SPSS (IBM Corp) to assess significant differences between pre and postnotes, which were matched at the patient level. Significance was set at .05. Mean scores per item were calculated for each patient to account for varying note counts. The Wilcoxon test, appropriate for paired and ranked data, is robust to nonnormality and small sample sizes [96].

Qualitative data were analyzed using thematic analysis with MAXQDA software (Verbi Software Ltd) [97]. An inductive-deductive approach was applied, following 6 steps: familiarizing with the data, generating codes, identifying themes, refining themes, defining themes in relation to research questions, and formulating key concepts. Initial codes and themes were discussed collaboratively until consensus was reached, and coherence was ensured. To ensure high-quality reporting, the Consolidated Criteria for Reporting Qualitative Research (COREQ) checklist was used (Multimedia Appendix 2) [98].

### Sample

#### Document Analysis: Sample and Sociodemographic Information

A total of 876 clinical notes were analyzed, consisting of 453 prenotes and 423 postnotes from 97 participating patients and 10 clinicians. The HCPs who wrote the clinical notes were, with the exception of one person, the same as those who were interviewed. Sociodemographic data could not be collected for one HCP, except for gender and professional group. The age of the HCPs ranged from 21 to 61 (mean 44.67, SD 12.01) years. The sample included an equal number of female and male participants (5/10, 50% each). The open notes were written by 5 (50%) psychiatrists, 4 (40%) psychologists, and 1 (10%) social worker. They worked either part-time (7/9, 77.78%) or full-time (2/9, 22.22%) in psychiatric outpatient clinics. The HCPs were evenly distributed across the 3 study centers (distribution: 4/3/3). Their clinical experience ranged from 2 to 31 (mean 13.33, SD 9.95) years.

Of the participating patients, 55.67% (n=54) were female, 34.02% (n=33) were male, and 1 person (1.03%) identified as nonbinary. Nine (9.28%) patients did not provide gender information. Patients ranged in age from 18 to 60 years or older (mean 40.79; SD 17.01). A total of 89.69% (n=87) of the participating patients identified themselves as White. Only 1 (1.03%) patient was identified as a person of color. Nine (9.28%) patients did not indicate their ethnicity. Four patients (4.12%) reported that they had a migrant background and did not speak German as their first language. In addition, 6 (6.19%) patients reported that their parents had a migrant background. Regarding employment status, 27 (27.84%) patients were employed, 9 (9.28%) were seeking employment, 34 (35.05%) were retired, and 16 (16.49%) were unable to work due to illness. A total of 11 patients (11.34%) did not provide information about their employment.

#### Interview Study: Sociodemographic Information

A total of 10 HCPs participated in the qualitative interviews. The sample included 3 (30%) senior physicians and 2 (20%) specialists in psychiatry, alongside 4 psychologists (40%) and 1 (10%) social worker. All participants were employed within a psychiatric hospital. All HCPs (10/10, 100%) worked in the psychiatric outpatient clinic. The participants’ professional experience ranged from 2 to 32 years, with a mean of 14.2 (SD 9.78) years. A substantial proportion of the participants (9/10, 90%) had psychotherapeutic training, including cognitive behavioral therapy (6/9, 67%), psychodynamic therapy (2/9, 22%), and client-centered therapy (1/9, 11%). Gender distribution was balanced, with 50% female (n=5) and 50% male (n=5) participants. The age of participants ranged from 26 to 61 years, with a mean age of 45.7 (SD 11.79) years.

## Results

### Overview

The findings of this mixed methods study demonstrate measurable changes in the language used within clinical documentation, as revealed through document analysis. These changes are further reflected within the qualitative results from interviews with HCPs, offering deeper insights into the processes of changes in documentation (practices) and the potential underlying reasons behind them.

### Quantitative Results

The number of prenotes per patient ranged from 1 to 28, with a mean of 4.67 (SD 4.22), while postnotes ranged from 1 to 23, with a mean of 4.36 (SD 3.66).

None of the items followed a normal distribution; therefore, a nonparametric test was applied. To assess differences between pre and postintervention documentation, a Wilcoxon signed rank test was performed. This test does not imply causal relationships, but rather indicates whether the observed differences are unlikely to have occurred by chance. Statistically significant changes were observed in 11 out of the 16 language characteristics examined. The results revealed significant differences for monoglossic, heteroglossic, comprehensible, incomprehensible, demeaning, resource-oriented, positive emotional, controlling, stigmatizing, personal, and factual language (Table 1).

**Table 1:** Pre and post comparison of linguistic features using a Wilcoxon signed rank test (876 notes on 97 patients). Ratings were performed on a 3-point Likert scale (1=not present, 2=neutral, 3=present).

Language style	Before opening notes			After opening notes			*z*score^a^		*P* value^b^		*r* value^c^
	Mean (SD)	Median	Mean (SD)		Median						
1. Monoglossic	2.74 (0.42)	3.00	2.57 (0.47)		2.71	–3.08		.002		0.313	
2. Heteroglossic	1.13 (0.29)	1.00	1.34 (0.45)		1.00	–3.99		<.001		0.405	
3. Comprehensible	2.47 (0.55)	2.50	2.73 (0.40)		3.00	–4.31		<.001		0.438	
4. Incomprehensible	1.69 (0.70)	1.50	1.43 (0.56)		1.14	–3.528		<.001		0.358	
5. Appreciative	1.58 (0.48)	1.50	1.57 (0.52)		1.50	–0.334		.74		0.034	
6. Demeaning	1.60 (0.50)	1.67	1.21 (0.34)		1.00	–5.75		<.001		0.584	
7. Resource-oriented	1.52 (0.51)	1.50	1.80 (0.65)		2.00	–4.04		<.001		0.410	
8. Deficit-oriented	2.40 (0.60)	2.50	2.31 (0.61)		2.40	–1.716		.09		0.174	
9. Positive emotional	1.48 (0.48)	1.38	1.59 (0.49)		1.64	–1.990		.047		0.202	
10. Negative emotional	1.96 (0.66)	2.00	1.86 (0.57)		2.00	–1.454		.15		0.148	
11. Empowering	1.61 (0.49)	1.67	1.72 (0.54)		1.75	–1.714		.09		0.174	
12. Controlling	1.94 (0.63)	2.00	1.69 (0.73)		1.50	–3.10		.002		0.312	
13. Stigmatizing	1.32 (0.49)	1.00	1.10 (0.23)		1.00	–4.34		<.001		0.441	
14. Destigmatizing	1.44 (0.52)	1.00	1.41 (0.50)		1.00	–0.551		.58		0.056	
15. Personal	1.07 (0.18)	1.00	1.40 (0.52)		1.00	–5.76		<.001		0.585	
16. Factual	2.95 (0.16)	3.00	2.73 (0.43)		3.00	–4.98		<.001		0.506	

^a^Standard score.

^b^Statistically significant.

^c^Effect size.

Considering the changes in median values, we can assume that monoglossic language (Premedian=3.00; Postmedian=2.71), incomprehensible language (Premedian=1.50; Postmedian=1.14), demeaning language (Premedian=1.67; Postmedian=1.00), and controlling language (Premedian=2.00; Postmedian=1.50) tend to decrease as a result of the intervention. Conversely, comprehensible language (Premedian=2.50; Postmedian=3.00), resource-oriented language (Premedian=1.50; Postmedian=2.00), and positive emotional language (Premedian=1.38; Postmedian=1.64) appear to have increased.

**Figure 1:**
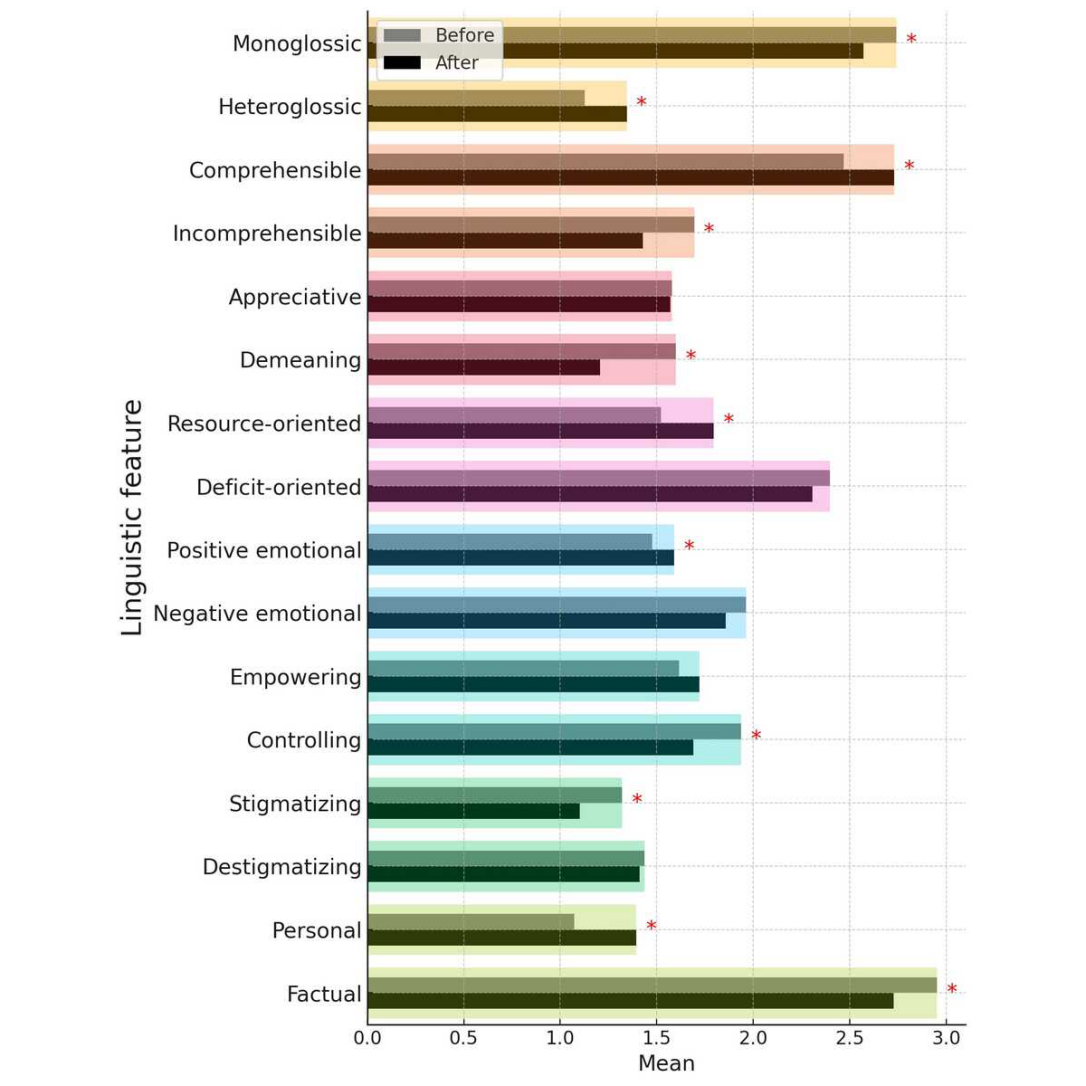
Changes in mean values of linguistic features. The asterisk (*) indicates a significant change in the Wilcoxon signed rank test.

Although statistically significant, 4 language characteristics—heteroglossic language (median 1.00), stigmatizing language (median 1.00), personal language (median 1.00), and factual language (median 3.00)—did not exhibit changes in median values before and after the intervention. However, changes in the mean values indicate an increase in heteroglossic (Premean 1.13; Postmean 1.34) and personal (Premean 1.07; Postmean 1.40) language, alongside a decrease in stigmatizing (Premean 1.32; Postmean 1.10) and factual (Premean 2.95; Postmean 2.73) language. For all significant changes in the Wilcoxon signed rank test, corresponding changes in the mean values can be observed, as shown in Figure 1.

### Qualitative Results

#### Changes in Documentation

**Figure 2:**
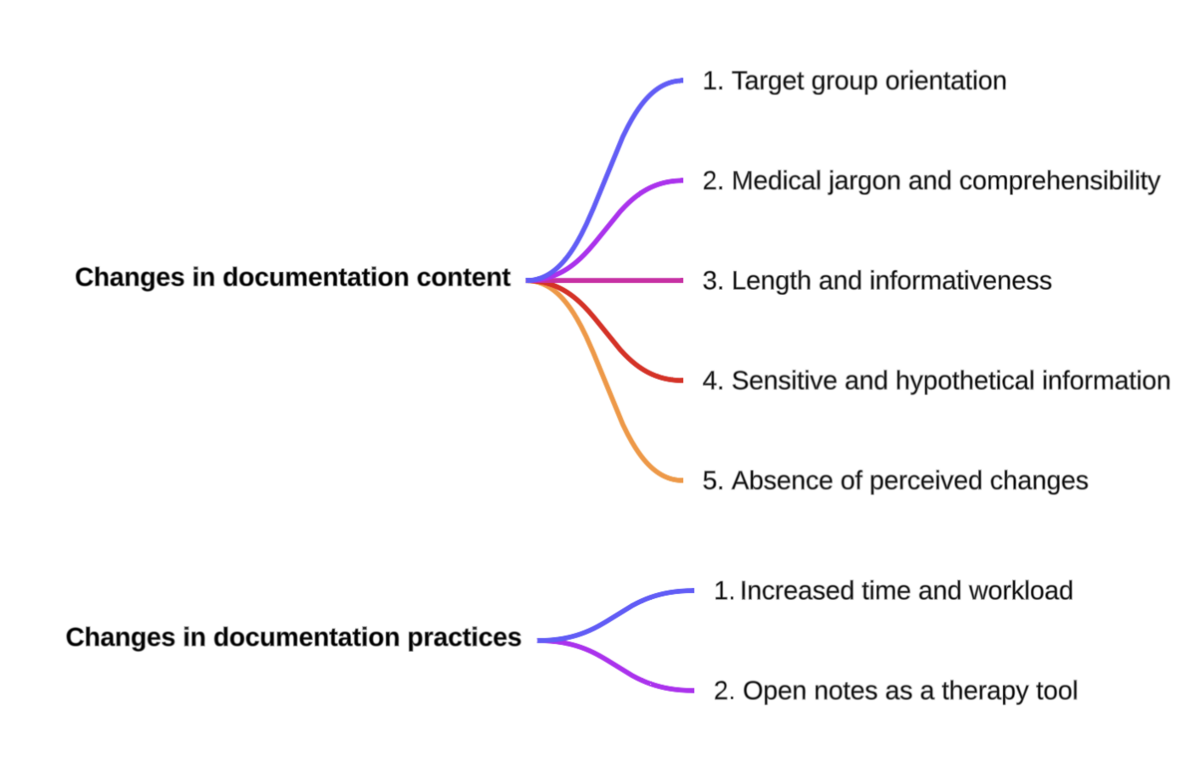
Overview of themes and subthemes identified in qualitative analysis.

The qualitative analysis revealed 2 overarching dimensions of change resulting from open notes (Figure 2): first, changes in the content of documentation itself, and second, changes in documentation practices, referring to how HCPs document. These changes are summarized below and then described in more detail in the subsequent subsections. Regarding the documentation content, multiple changes occur due to open notes. One major change concerns the intended audience of the notes. Whereas notes were previously primarily directed toward internal and external colleagues, open notes are consciously addressed to patients and, where applicable, their care partners. This shift is accompanied by adaptations in language: generally, HCPs now write in a more accessible manner, using less medical jargon. Consequently, open notes tend to be more detailed and contain more explanations compared with traditional closed clinical notes. However, HCPs typically take care to omit or paraphrase sensitive and hypothetical information. Conversely, some HCPs report that their documentation has not significantly changed despite the introduction of open notes. Concerning changes in documentation practices, HCPs report a noticeable increase in workload. Nonetheless, this phenomenon appears to be mitigated, at least in part, by the novel opportunities that open notes present as a therapeutic instrument.

The reported results of the qualitative analysis are illustrated with exemplary quotes from the interviewees. The included quotes are labeled as follows: Participant 01-10, paragraph in the interview transcript, and profession.

#### Target Group Orientation

HCPs (n=9) reported tailoring their documentation to different target groups, including colleagues, patients, family members, or health insurance companies. This audience-specific consideration influenced how clinical notes were composed as open notes. HCPs highlighted a distinct contrast between documentation intended for colleagues (closed notes) and documentation shared with patients (open notes). Respondents characterized notes for colleagues as concise, used technical terminology, and focused on risk factors. In contrast, HCPs reported composing more detailed and resource-oriented clinical notes, when they were shared with patients. Furthermore, they tried to avoid specialized language and jargon in the open notes. These differences are exemplified by the following quotes:

You can use shorter technical terms for colleagues because they interpret and contextualize them differently. They have a different level of knowledge that patients do not have. So the treatment context is something that I try to explain differently when I know that patients might be reading along.

participant 05, 9, social worker

I would still discuss certain difficult situations with the patient. But if I were to present a potential crisis situation to the physician in the course of treatment, I would document it differently. [...] If I'm writing for the patient, it's always more resource-oriented, whereas if I'm writing for colleagues, I would emphasize potential risks more.

participant 04, 10, psychologist

The need for patient orientation compels HCPs to adopt the perspective of the patient and incorporate it into the documentation process. Target group orientation thus forms the foundation for patient-centered documentation, interlinking with all subsequent subtopics, which explore specific aspects of documentation adjustments in greater depth.

#### Medical Jargon and Comprehensibility

HCPs (n=8) expressed a commitment to making their documentation more comprehensible for patients with access to open notes. A core component of this effort involved adapting the language of the documentation to align with patients’ own expressions (n=4), as demonstrated by the following comment:

I did try to adapt to the language of the patients. For example, when the documentation involved references to relatives, instead of writing something like, 'Patient reports about his mother,' I started using the specific wording the patient used for the mother. This is something I didn’t pay attention to before and would have just written in my usual way.

participant 02, 22, psychologist

This more comprehensible language style includes that HCPs (n=6) shift away from telegraphic, bullet-point notes in favor of more detailed prose. Several HCPs (n=7) also intentionally minimized or rephrased technical terms or medical jargon to enhance accessibility for patients. One HCP says:

I try not to use technical terms, or if I do, I briefly explain them in parentheses. (...) Also, I tend to write the psychopathological findings in a more descriptive and less technical style, integrating them into the text. I also write more complete sentences—subject, predicate, object—rather than using a telegraphic style. It’s more like proper prose, I’d say.

participant 03, 18, psychiatrist

Conversely, a few HCPs (n=4) maintained a focus on clarity and factual precision in their open notes, ensuring medical jargon was avoided without compromising the clinical accuracy of the documentation:

I often wrote more clearly. I actually noticed that I moved away from using very clinical terms.

participant 02, 10, psychologist

#### Length and Informativeness

The length and informativeness of open notes were closely tied to their comprehensibility. Efforts to simplify language and avoid abbreviations or technical jargon naturally resulted in longer entries due to more detailed descriptions and explanations. One HCP reports:

The text became a bit longer. As mentioned, I avoid abbreviations, avoid technical terms, and try to use more understandable language.

participant 08, 28, psychiatrist

The increased length stemmed from 2 primary factors. First, many HCPs (n=7) enhanced the level of detail and completeness in their documentation, as the following quote illustrates. In some cases, HCPs even provided a verbatim account of what was discussed during consultations.

I tried to cover as much of what we discussed as possible, so I didn't leave anything out. [...] So I tried to mention as much as possible of what we discussed. I paid attention to details, so completeness in that sense.

participant 09, 12, psychologist

As already indicated in the previous quote, some HCPs (n=5) incorporated more explanatory content about treatment-related matters, such as medication changes, to improve patient understanding. This additional content would be unnecessary for their colleagues—as described in the following comment—and is the second reason for the prolongation of the documentation.

For something like increasing a medication dose, I would normally just write: 'Dose increased’. Period. Because I assume a colleague would check the medication record for details. But that approach doesn't work here-it's the only documentation for the patient. So it's important to specify what medication was increased and to what dose.

participant 01, 12, psychiatrist

However, a small number of HCPs (n=4) reported using a more concise, focused documentation style with the implementation of open notes. For these HCPs, this approach results in more compact documentation, as stated in the following quote:

I try to express myself as clearly and concretely as possible without rambling or including unnecessary details. I aim to stay focused and write only what’s most important.

participant 09, 6, psychologist

#### Sensitive and Hypothetical Information

The implementation of open notes poses a particular challenge for HCPs in managing sensitive or hypothetical information. Typically, such information would be shared among colleagues in closed notes to highlight potential risks or diagnostic hypotheses. However, when patients have access to their clinical notes, additional considerations and adjustments are required.

#### Sensitive Information

The interviews show that, depending on the nature of the sensitive information, HCPs consider whether to withhold and omit it from the documentation altogether (eg, in the case of violence or abuse) or to describe the sensitive information openly—but in a careful and respectful manner (eg, regarding relapse, sexual dysfunction, dementia, or aggressive behavior). More than half of HCPs (n=6) were aware that third parties—referred to as secret readers (eg, family members, internal and external colleagues)—may access the open notes, regardless of the patient’s intent. This awareness influences how HCPs document sensitive information, often leading to cautious, nonspecific language or omissions to protect patients. Some HCPs reported using generic terms or keywords for sensitive information even before open notes were implemented:

Sensitive information that others really shouldn't know and that should explicitly remain in the protected therapeutic space - I don't document that explicitly. I just touch on it with a broad term. That hasn't changed with open notes.

participant 10, 19, psychologist

Other HCPs (n=5) argue that nothing of importance should be omitted and that even sensitive information should be described openly. However, how openly HCPs documented this information depended on the stability of the patients and the nature of the information.

I addressed the issues that came up, even if they were sensitive. I think it's important not to leave out anything that was part of the discussion.

participant 09, 20, psychologist

More than half of the HCPs (n=6) were aware of the potential existence of secret readers. After some consideration, HCPs came to the conclusion that it is the patient's responsibility to decide who should have access to their treatment records.

I know anyway that much of what I discuss with my patients is passed on to third parties anyway. [...] I have written it in such a way that it is OK for the patient. [...] There was no legal guardian with my patient, there was no one to look at it. They were all very independent patients. Yes, and that's why I didn't think about it. And that's why I thought to myself: Well, if they're going to show it to somebody, they're going to show it with determination and that's OK.

participant 02, 36, psychologist

#### Hypothetical Information

Regarding hypothetical information, HCPs primarily discussed how they document suspected diagnoses. Some HCPs (n=3) reported that they do include hypotheses in their notes, ensuring that they clarify both the HCP’s and the patient’s perspectives when they diverge.

When I include a hypothesis, I clearly state that it’s from my professional perspective. I emphasize that, from the patient’s perspective, things might appear a certain way, but from mine, it could be different. It’s important to make clear that these are hypotheses from my perspective.

participant 03, 32, psychiatrist

Other HCPs (n=5) indicated that they discuss their professional hypotheses directly with patients and only include hypothetical information in the documentation if it has already been addressed in a personal conversation. One HCP comments on this topic as follows:

Regarding hypotheses, I might document them if I’ve already discussed them with the patient. But I wouldn’t write something entirely new, like ‘I think there’s an underlying psychosis’ if the patient hasn’t heard about it before. I’d always address it in a conversation first and then document it accordingly.

participant 06, 16, psychiatrist

#### Information Blocking

A small number of HCPs (n=3) stated that they deliberately exclude certain types of information from open notes for various reasons, including concerns about potential negative impacts on the patient or their treatment. HCPs omit personal impressions, such as countertransference feelings, treatment-related concerns, or sensitive topics like suicidality, to mitigate potential harm, as the following quotes demonstrate:

There are things I leave out for patients, such as certain concerns about the future course of treatment — things that might highlight risks or suggest a potential crisis. I’d omit those kinds of concerns.

participant 04, 10, psychologist

#### Absence of (Perceived) Change

While the majority of HCPs' statements include explicit (and implicit) references to a change in documentation style following the implementation of the Open Notes, all 10 HCPs reported that the content of their documentation has remained unchanged—at least to some degree. A few stated that they made no content adjustments to accommodate patients, while others noted that specific aspects or areas of their documentation (eg, handling sensitive information) remained unchanged. The subsequent citation elucidates the absence of change.

I don’t think my documentation has changed. The content has stayed the same.

participant 05, 22, social worker

Half of the HCPs (n=5) also indicated no alteration to the language and form of the open notes in comparison to previous closed notes, maintaining their personal documentation style. For some (n=2), this meant continuing to use medical terminology without translating or simplifying it for patients. Others (n=3) emphasized that they had already used respectful and patient-oriented language in their documentation before open notes were introduced, making further changes unnecessary.

I consciously decided to continue documenting as I always have.

participant 10, 7, psychologist

And otherwise, I’ve always aimed for respectful documentation that patients can engage with. I’ve done that anyway because, sometimes, doctors read aloud what I’ve written to the patients. So, not much has changed in that regard.

participant 05, 22, social worker

#### Changes in Documentation Practices

The content and formal adjustments described previously illustrate that HCPs are engaging more thoughtfully with the creation of clinical notes, striving to adopt patients’ perspectives and implement a more patient-centered approach. Beyond these modifications, HCPs reported additional changes in their documentation practices. Approximately one-third of HCPs (n=3) explicitly mentioned being more mindful and reflective during the documentation process:

Because I always tried to adopt the patient’s perspective. I didn’t want to sugarcoat or downplay anything, but I wrote much more reflectively and with greater mindfulness.

participant 10, 13, psychologist

Some HCPs (n=4) made a conscious effort to frame their clinical notes in a more resource-oriented manner when patients could access them. Others aimed to ensure their documentation was as nonjudgemental as possible. They found that writing concretely and factually supported this effort. The following quotes illustrate these approaches:

Yes, so what has changed positively is that I think my documentation has become more detailed, more helpful to patients and also more appropriate. It has become, as I said, more future- and resource-oriented and more accessible.

participant 03, 38, psychiatrist

Well, I've tried to write it without judgement as far as possible, but to take what she reported as fact and to reproduce the content.

participant 09, 8, psychologist

#### Increased Time and Workload

All HCPs (n=10) observed that creating open notes required more time and effort compared with writing closed notes. Each of the 10 HCPs described putting more thought and effort into writing the open notes, thereby increasing their workload:

It just wasn't like that before and that leads to [...] a bit more stress, a bit more work and but also more self-reflection.

participant 03, 28, psychiatrist

For slightly more than half of the HCPs (n=6), this increased self-reflection came with a notable time burden, which was particularly difficult to accommodate within the tight schedules of clinical work.

It’s not theoretically much more work, but just opening another program, copying things over, and especially crafting documentation that is empathetic, comprehensible, and essentially also a therapeutic intervention - it’s sometimes too much. I usually document on the side, but this really pushes the limits. I need to set aside three to four more minutes to write something good. And if I have ten patients using Open Notes in a row, that’s, let’s say, five minutes per patient - 50 minutes more work per day, and that adds up.

participant 03, 8, psychiatrist

Another key challenge faced by most HCPs (n=8) was navigating the technical demands of using a patient portal for open notes. Many criticized the dual documentation process, which required inputting data into both internal systems and the patient portal, as well as completing a 2-factor authentication process; calling for better integration of open notes into existing systems to make them more practical for everyday clinical use.

I found the two-factor authentication more challenging since I always needed my phone. There were times I had to go back and get it. I did it for the study, but in everyday life, I probably wouldn’t have bothered.

participant 01, 28, psychiatrist

#### Open Notes as a Therapeutic Tool

The interviews reveal that most HCPs (n=8) perceive open notes not only as a documentation tool but also as a therapeutic intervention. HCPs identified three primary functions of open notes as a therapeutic tool as follows: (1) expanding (the therapeutic space), (2) validating, and (3) structuring.

First, open notes can extend the therapeutic space into the digital realm, facilitating patient engagement beyond the in-person session. This occurs through different postsession interactions with the open notes: patients can revisit, remember, and reflect on what was discussed, thereby reinforcing the therapeutic session (memory function). Additionally, some HCPs use open notes to assign therapeutic tasks (homework) or other responsibilities, such as coordinating with external providers.

I notice that some patients read the notes regularly, even if they don’t comment on them, but they say it makes them think deeply about how I perceive them or what I’ve written. [...] It extends or shifts the therapeutic conversation into this digital space and also beyond the actual appointments. It allows us to revisit concerns, conflicts, or evaluations about their social reality or themselves in subsequent sessions.

participant 03, 36, psychiatrist

Yes, I’ve used it as a therapeutic tool in the sense that it serves as a reminder - a summary of what was discussed. Often, a lot is forgotten during a medical encounter. It’s also a way to validate the patient and provide clarity.

participant 07, 41, psychiatrist

As the previous quote suggests, open notes can also play a validating role. Patients can read what their HCPs have written, share their opinions, provide feedback, and correct inaccuracies. This dynamic can enhance the quality of the clinical documentation.

Since patients read along, it acts as a form of quality control. Even if they don’t have the medical expertise, they can still say whether what’s written about them is accurate or not. They’re the best judge of that.

participant 07, 67, psychiatrist

Finally, open notes aid some HCPs organizing their thoughts and reflecting more carefully on the treatment (internal organizing function). Knowing that patients will read their documentation encourages a more deliberate, structured documentation process, thereby enhancing their own clarity and the therapeutic approach.

The process of documenting helps organize the thoughts, interactions, or what the patient has said into a structured framework. It allows me to better order the conversation.

participant 03, 36, psychiatrist

## Discussion

### Integration of Quantitative and Qualitative Findings

**Figure 3:**
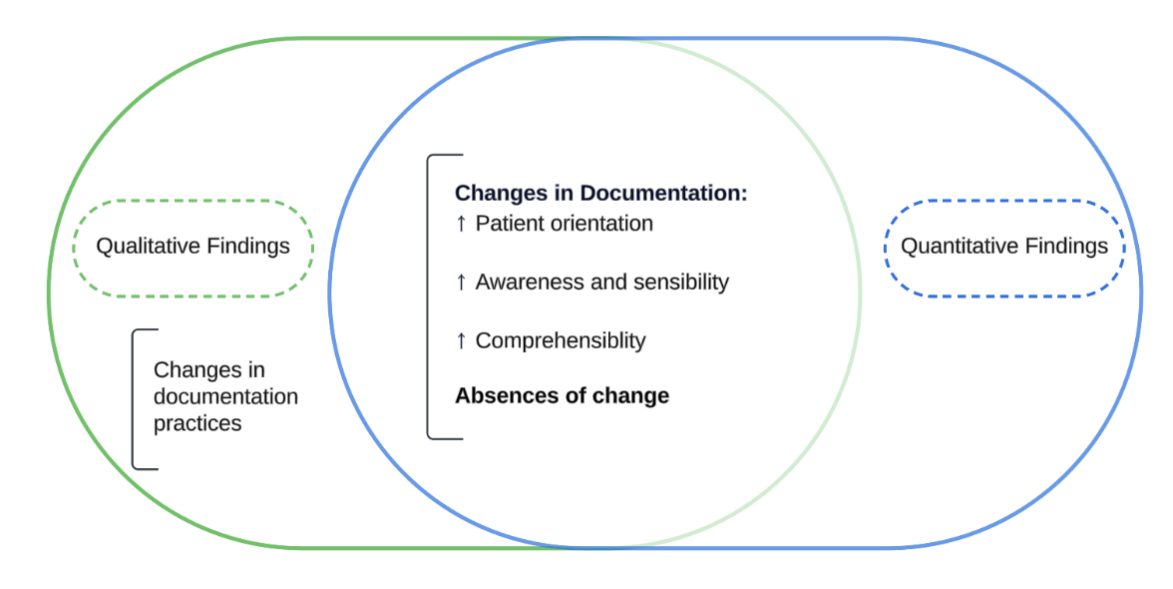
Integration of quantitative and qualitative findings.

A comparative analysis of clinical notes before and after the implementation of open notes revealed significant changes in the language style of the documentation. Integrating qualitative and quantitative findings, as illustrated in Figure 3 and elaborated on in the following subsections, provides added value beyond what either approach could offer alone. The qualitative data reinforce the quantitative results and offer nuanced insights into the context and possible reasons behind the observed changes—or lack thereof. This complementary perspective enhances the depth and interpretability of the findings, demonstrating how a mixed methods approach can yield a more comprehensive understanding of complex documentation practices.

The quantitative and qualitative findings indicate that clinical notes written after open notes implementation are generally more resource-oriented and positive in tone, which aligns with the existing evidence [20]. HCPs actively try to emphasize patients’ strengths, progress, and abilities and to reframe psychological crises as opportunities for growth. Concurrently, the quantitative results show a significant reduction in monoglossic, demeaning, and controlling language, suggesting that documentation now increasingly incorporates not only the voices and perspectives of HCPs but also considers those of patients. This includes a conscious effort to avoid hierarchical or paternalistic statements and to refrain from judgmental language. The observed decline in controlling and demeaning language in the quantitative results may also reflect heightened awareness among HCPs for their patients’ needs, particularly when handling sensitive or potentially retraumatizing content. This is also reflected in the interviews and other existing research, where HCPs report being increasingly cautious and considerate when handling sensitive and hypothetical information that may have a stigmatizing, retraumatizing or harmful impact on patients—for example, in cases involving domestic violence [30,31,99,100]. Furthermore, the quantitative results indicate an increased patient orientation in the documentation after open notes implementation, which is also reflected in the qualitative findings. Clinicians emphasize keeping the patient “in mind” when writing notes, addressing them at least implicitly, and increasingly avoiding impersonal, telegraphic, or fragmented styles of documentation. Considering the qualitative analysis, it can be assumed that while HCPs tend to adopt a more personal documentation style with open notes, many still place great importance on maintaining precision and clarity in their documentation—a finding that is also supported by existing evidence [20,35,36,101]. Moreover, both subjective and objective changes suggest a shift toward more comprehensible language, which often involves minimizing the use of medical jargon, providing more detailed explanations, and tailoring the note content to patients’ needs. This shift frequently leads to (slightly) longer notes than those written before the adoption of open notes. These observations are consistent with the existing body of evidence on the effects of open notes and transparent communication practices [32-34].

Alongside these reported and observed changes, both the qualitative and quantitative findings indicate that certain aspects of documentation remain unchanged. For instance, some language characteristics, such as appreciative, empowering, and destigmatizing language, as well as negative emotionality, do not show measurable alterations. Based on the qualitative findings, it can be assumed that most HCPs were already documenting in a respectful and appreciative manner before the introduction of open notes, which explains the lack of significant changes in the objective results. Regarding the persistence of a deficit-oriented language in the quantitative results, it appears that while HCPs tend to adopt a more resource-oriented style with open notes, they still need to maintain a certain degree of deficit orientation to justify services and reimbursement within the German health care system. Qualitative results further suggest that these changes extend beyond the notes themselves, influencing the documentation practices of HCPs: HCPs reported adopting a more patient-centered approach to their documentation. While initial studies suggest that digital health records and patient portals may promote more patient-centered care, the existing body of research remains limited, particularly with regard to the implementation and impact of open notes [102-104].

As reported in other studies, interviewed HCPs also described safeguarding sensitive or hypothetical information by summarizing it in more general terms, a practice many had already adopted prior to the implementation of open notes [20]. Notably, despite stylistic adjustments, all interviewed HCPs emphasized that the substantive content of their documentation remained unaltered, with no critical information omitted. This contrasts with previous studies indicating that the content of open notes may change when clinicians withhold or censor certain information [20]. However, open notes concomitantly engendered novel opportunities to extend the therapeutic engagement into the digital realm.

### Rethinking Clinical Documentation: Opportunities, Challenges, and Implications

The findings from both qualitative and quantitative analyses suggest that open notes can lead to changes in the language, word choice, and style of clinical documentation. Consistent with prior research, clinical notes appear to become more patient-oriented, comprehensible, and empathetic following the implementation of open notes [24,105]. This transformation unveils novel opportunities to strengthen the connection between patients and HCPs: As the act of composing open notes fosters deeper attunement to patients’ perspectives, patients who read these notes often report feeling seen and understood [106,107]. This mutual understanding has the potential to enhance the therapeutic alliance, which is a key factor in psychotherapy [108].

Nonetheless, these benefits are not without challenges. HCPs’ concerns about increased workload are validated by findings that highlight the additional time required to create open notes. These findings do not align with studies on the experiences of HCPs with open notes, which report that—despite initial concerns—their workload tends not to increase over time [35,109-113]. In the tightly scheduled clinical environment, where clinicians often have only a few minutes per patient, such additional demands may quickly provoke resistance among HCPs [16]. Overcoming these barriers requires structural changes that facilitate the seamless adoption of open notes. This study identifies three critical needs: (1) streamlined integration of open notes into existing clinical documentation systems, (2) a reconceptualization of clinical documentation itself, and (3) documentation training and guidelines for HCPs. Structural changes seem necessary to ensure that open notes are seamlessly integrated into the HCPs’ workflows. Integration must eliminate the inefficiency of dual systems and redundant authentication processes. Open notes should be embedded within the primary documentation platform to avoid workflow disruptions.

Beyond technical integration, the conventional view of clinical documentation as a tool for only professional communication or a personal reference for HCPs themselves warrants reevaluation [31,37,114]. For some HCPs, adapting open notes to ensure they are understandable to patients (eg, by using lay language) entails a perceived loss of objectivity, accuracy, and clinically relevant detail, potentially compromising effective interprofessional communication [32,33,101]. In particular, hypothetical information such as tentative differential diagnoses, personal assessments, or observations, as well as medical jargon, may be omitted from open notes under these circumstances, which can negatively affect multidisciplinary communication [38,39,114,115]. With ongoing psychiatric reforms since the 1970s and the growing emphasis on patient participation in treatment decisions (shared decision-making), the ownership and intended audience of clinical notes have become pertinent questions [116,117]. Clinical documentation emerges from a collaborative therapeutic process involving both HCPs and patients. This collaborative nature challenges the notion of exclusive authorship and raises important considerations about ownership and audience. It highlights the need to examine how clinical notes are attributed and for whom they are primarily written.

Reframing clinical notes as shared, dynamic therapeutic tools could shift HCP perceptions of potential challenges that come with open notes [37]. Rather than fearing that open notes will confuse or distress patients or dilute professional rigor, HCPs might embrace documentation as a creative, participatory endeavor. This paradigm shift has the potential to change power distributions between HCPs and patients, fostering synergies that reflect the transformative potential of collaborative care and underscoring the notion that (even in clinical documentation), change can catalyze growth [40,118]. For these positive outcomes to materialize, patient-centered and empathetically composed notes are essential. To cultivate such open notes, HCPs require the time and mental space to empathize with their patients’ perspectives, language, educational backgrounds, and emotional concerns.

Beyond structural changes, specialized training programs and workshops are essential to equip HCPs with the skills to compose open notes that are both patient-centered and efficient. Integrating such training into medical and health care education could prepare future HCPs early on. Additionally, standardized documentation guidelines should be developed to provide clear frameworks for patient-centered documentation, particularly in challenging scenarios such as handling sensitive information. An initial contribution in this regard has been made by Vanka et al [104], who proposed 10 documentation guidelines.

Advances in generative artificial intelligence (AI) and ambient AI technologies are already being integrated into clinical documentation [119], offering potential benefits such as increased efficiency, rapid note generation, and adaptability to different tones and literacy levels. The use of these tools to create patient-facing notes that are both accessible and empathetic remains an emerging area of exploration [120,121]. However, challenges such as misinformation (AI “hallucinations”), biases, privacy risks, and potential harms must be carefully considered. Moving forward, it will be essential to educate clinicians on the adoption of these tools while ensuring they maintain oversight and actively edit AI-generated documentation to uphold accuracy and patient safety [122].

### Strengths and Limitations

To our knowledge, this is the first study to examine objective documentation changes in a mental health context using robust nonparametric analyses, addressing a significant gap in the literature. Nevertheless, the study has various limitations: Since comparing ranks between pre and postnotes was involved, we used the Wilcoxon signed rank test. While a *t* test might offer greater power, the Wilcoxon signed rank test is particularly suited for nonnormally distributed data, providing robust and reliable results for paired observations. Additionally, the study focused on the linguistic characteristics of clinical notes rather than examining quantitative parameters such as word count, note length, or n-grams [26]. Moreover, standardized linguistic analysis tools like the linguistic inquiry and word count were not applied, which could reduce objectivity and traceability, potentially leading to subjective bias [41]. However, the raters underwent extensive training in the evaluation of linguistic features to minimize these effects. Furthermore, not every clinical note was rated by 2 independent raters, which may limit the objectivity and replicability of the findings. However, these limitations were mitigated through a structured training process, a detailed coding manual, and a thorough precoding phase. These measures contributed to maintaining a high level of consistency across the full dataset.

A limitation inherent to clinical note analysis is the challenge of determining whether HCPs altered their original documentation intent by softening, rephrasing, or omitting content—insights known only to the HCPs themselves. It is possible that clinicians documented differently than they normally would, knowing that their documentation was being observed by both patients and the research team [123]. To mitigate this potential Hawthorne effect, a prolonged observation period of 12 months was chosen. Prior research suggests that over time, individuals tend to return to their habitual behavior as they acclimate to being observed or to a new intervention setting [124]. Furthermore, we triangulated both quantitative and qualitative data sources to examine whether observed changes were consistent across different types of data. This allowed us to assess whether changes appeared only in self-reported experiences or were also reflected in documentation itself. Regarding participant sampling, a potential self-selection bias may exist, as HCPs and patients who agreed to participate might exhibit higher levels of digital literacy and a more positive disposition toward transparent documentation practices. Nonetheless, based on the study team’s observations, particularly concerning patients, the digital literacy levels appeared diverse.

Regarding a potential recruitment bias, the purposive sampling and inclusion criteria focused on patients with severe mental illness who were undergoing outpatient treatment and able to provide informed consent. Patients with acute suicidality, psychosis, or severe cognitive impairments were excluded. Recruitment took place via their treating HCPs, who may have tended to select patients perceived as more stable, cooperative and digitally literate. With respect to HCPs, it is possible that the study team predominantly recruited individuals who were already comfortable with digital tools, potentially limiting the diversity of clinical perspectives. These factors should be considered when interpreting the results, as they may limit the generalizability of our findings to more stable, digitally engaged patients and digitally literate HCPs. The digital literacy was not formally assessed with standardized instruments such as the eHealth Literacy Scale [125]. However, both the study team and participating HCPs made a deliberate effort to include a diverse range of participants in terms of attitudes toward digital health tools and digital literacy.

Furthermore, the study was conducted in 3 outpatient clinics within a single rural region in Germany, involving predominantly White participants. As a result, the generalizability of the findings is limited, particularly with regard to more diverse populations, urban or inpatient mental health settings, and other areas of care such as somatic medicine. While the sample was drawn from a single mental health setting, the insights gained are particularly relevant for understanding how open notes affect psychiatric documentation (practices)—a field where transparency and patient engagement are critical. In addition, some patients’ notes, including those with severe mental illness or those who were suicidal, were omitted, and this may have biased our findings.

Future research endeavors should explore whether and how the traditional conceptualization of clinical notes as a working tool exclusively for and between professionals can be broadened, allowing notes to be reconceptualized as a participatory therapeutic tool. In addition, we emphasize that further studies are focused on examining objective changes to clinical documentation following open notes, not only in mental health care but in other medical specialties.

### Conclusions

This study demonstrated that open notes implementation in 3 German outpatient centers led to both objective and perceived changes in clinical documentation within our sample. Significant changes were observed in 11 language characteristics, including monoglossia and heteroglossia, comprehensibility and incomprehensibility, demeaning and resource-oriented language, positive emotionality, control, stigmatization, personalization, and factuality. Clinicians in this study adopted a more patient-friendly writing style, and contrary to initial concerns, they did not omit critical information but reframed it to improve patient understanding. Participants highlighted the need for structural support, such as additional time for documentation, to facilitate the transition to patient-oriented note-writing.

## Appendix

Multimedia Appendix 1 Definitions of language characteristics.

Multimedia Appendix 2 Consolidated Criteria for Reporting Qualitative Research (COREQ).

## Abbreviations

AI: artificial intelligence
COREQ: Consolidated Criteria for Reporting Qualitative Research
HCP: health care professional
ICD-10: International Statistical Classification of Diseases, Tenth Revision

## Data Availability

The datasets generated or analyzed during this study are not publicly available, but are available from the corresponding author on reasonable request.
