# Supplementary material for: Changes in Documentation After Implementing Open Notes in Mental Health Care: Pre-Post Mixed Methods Study
Source: J Med Internet Res. 2025 Sep 3;27:e72667. doi: 10.2196/72667 (PMC12444227; doi:10.2196/72667)
Supplement: Multimedia Appendix 1 [file jmir_v27i1e72667_app1.pdf]

List and definitions of the language characteristics

Main category	Subcategory	Description / Definition	Scientific sources
**Monoglossic - Heteroglossic**		<u>Monoglossic/unanimous:</u><br>Formulations that only include the author's voice and ignore the multitude of alternative views and voices that are likely to be at play in the current communicative context.<br><br>Example - Monoglossic:<br>A. The patient's behavior is aggressive.<br>B. The patient is in a good mood.<br><br><u>Heteroglossic / polyphonic:</u><br>Formulations that make it clear that the statements made are only one position within a multitude of possible alternative positions and points of view.<br><br>Example - Heteroglossic:<br>A. The patient's behavior can be interpreted as aggressive / There are indications of aggressiveness in the patient's behavior.<br>B. The patient states that she is in a good mood. / The patient gives the impression of being in a good mood.	[1–3]
**Comprehensible - Incomprehensible**		<u>Comprehensible language:</u><br>**Simple**: Use of familiar words and short, simple sentences. Vivid language style. Foreign words are explained. **Structured, organized**: The text is clearly laid out and the structure is easy to follow. **Short and concise** formulations.<br><br><u>Incomprehensible language:</u> **Complicated***:* Use of long and convoluted sentences. Text not very descriptive. Many foreign words are included (complicated). **Unclear, incoherent***:* There is no clear structure or common thread. Text appears disjointed and confused. **Verbose**: The text is (too) detailed. It contains a lot of irrelevant information.<br><br>(based on the Hamburg comprehensibility model)	[4–21]

Main category	Subcategory	Description / Definition	Scientific sources
	**Technical terms & abbreviations**	Medical terminology, foreign Latin words, medical jargon, abbreviations not understandable for the Pt	
**Appreciative - Demeaning**		<u>Appreciative language:</u><br>Respectful, understanding, accepting and compassionate-empathetic formulations. The writing is formulated in a perceptive/observational way. Moral value judgments, comparisons and stereotypes are avoided. An attempt is made to understand the patient's personal feelings and behaviors precisely and sensitively.<br><br><u>Demeaning language / disrespectful language:</u><br>Patients are evaluated and judged in formulations (possibly even stigmatized). There is no empathic change of perspective into the patient's lifeworld.	[22–29]
**Resource orientation - Deficit orientation**		<u>Resource orientation:</u><br>Patients' past and present strengths, resources, successes, potentials and abilities are recognized, encouraged and positively reflected in the documentation. Personal processes, growth and development of the patient are at the forefront of the documentation. (The psychological crisis is seen as an opportunity for development and growth).<br><br><u>Deficit orientation:</u><br>The documentation is mainly symptom-oriented. Resources and strengths are ignored. Negative basic assumptions regarding the diagnosis, prognosis and possible treatment options ("myth of incurability") as well as a deficient view of patients with regard to their diagnosis and the corresponding psychopathology dominate.	[6,26,30–37]
**Positive emotional - Negative emotional**		Words are used that express a quality of the six most important emotion types (love, joy, surprise, anger, sadness and/or fear).	[4,9,38–42]

Main category	Subcategory	Description / Definition	Scientific sources
		Positive emotion words:<br>**Love**: adoration, affection, love, tenderness, sympathy, attraction, care, tenderness, compassion, sentimentality<br>**Joy:**<br>- Amusement, bliss, happiness, cheerfulness, cheerfulness, joy, delight, pleasure, joy, happiness, jubilation, elation, contentment, ecstasy, euphoria<br>- Enthusiasm, eagerness, excitement, thrill, exhilaration<br>- Satisfaction, pleasure<br>- Pride, triumph<br>- Enthusiasm, hope<br>- Ecstasy, rapture<br>- Relief<br><br>Neutral emotion words<br>**Surprise**: amazement, surprise, astonishment<br><br>Negative emotion words<br>**Anger**:<br>- Anger, rage, indignation, fury, wrath, hostility, ferocity, bitterness, hatred, abhorrence, contempt, malice, vindictiveness, aversion, resentment<br>- Disgust, revulsion, contempt<br>- Envy, jealousy<br>- Pain<br>**Sadness**:<br>- Agony, suffering, pain, fears<br>- Depression, despair, hopelessness, gloom, dejection, sadness, unhappiness, grief, sorrow, woe, misery, melancholy<br>- Dismay, disappointment, dissatisfaction<br>- Guilt, shame, regret, remorse<br>- Alienation, isolation, neglect, loneliness, rejection, homesickness, defeat, dejection, insecurity, embarrassment, humiliation, insult<br>- compassion, sympathy<br>**Fear**:	

Main category	Subcategory	Description / Definition	Scientific sources
		- Alarm, shock, fear, horror, terror, panic, hysteria, humiliation<br>- Anxiety, nervousness, tension, uneasiness, worry, distress, fear	
**Empowering - Controlling**		Autonomy-responsive / empowering:<br>The documentation clearly shows that patients are actively involved in treatment planning and therapy decisions and that communication with them is transparent. Promoting the development of self-efficacy and personal responsibility are at the heart of treatment. Patients are enabled to maintain their autonomy and dignity (empowerment approach). The focus of treatment is on the patient's individual goals and wishes for change. It is assumed that patients' symptoms and behaviors have "good reasons" (functionality).<br><br>Controlling:<br>It is clear from the wording that a medical, symptom-oriented model of illness prevails, in which patients are urged to relinquish responsibility in the patient role (perpetuation of a paternalistic approach). Patients are not actively involved in therapy decisions and treatment planning. Treatment is automatically aligned and assessed based on symptoms. The documentation shows simplifications, stigmatizations, generalizations and categorizations.<br>Violent situations, incapacitation and compulsory treatment (restraint, compulsory medication, admission under the PsychKG, no exit), (strict/punitive) consequences for alleged misconduct or strict exit regulations may be described.	[29,30,43–48]
**Stigmatizing - Destigmatizing**		Stigmatizing: The shared documentation contains derogatory terms that refer to the somatic or mental condition of the patient (such as "fat", "disturbed", ...)	[49–51]

Main category	Subcategory	Description / Definition	Scientific sources
		Destigmatizing: In the shared documentation, potentially stigmatizing and shameful topics are addressed and described empathetically. It is not a matter of omitting or concealing certain topics. Rather, the practitioners are able to address sensitive topics with sensitivity without shaming the patient.	
**Personal - Factual**		Factual:<br>The documentation describes the facts in a purely factual and objective manner. Possibly written in a "telegram style". The documentation appears to have been written (as a reminder) for the authoring practitioner or (as a means of communication) for other colleagues. It is not addressed to the patient.<br><br>Personal:<br>The documentation was written with the patient "in mind". The patients are included in the writing and implicitly addressed.	[4,6,14,16,17, 20,21,52–61]

Validated metrics and frameworks that served as the basis for deriving the language characteristics:

**Monoglossic - Heteroglossic**	Coh-Metrix [62]
**Comprehensible - Incomprehensible**	Flesch-Kincaid Readability Tests [63]
**Appreciative - Demeaning**	Appraisal Theory [64]<br>Linguistic Inquiry and Word Count [65]
**Resource-oriented - Deficit-oriented**	Appraisal Theory [64]<br>Linguistic Inquiry and Word Count [65]
**Positive emotional - Negative emotional**	Linguistic Inquiry and Word Count [65]
**Empowering - Controlling**	Politeness Theory [66]<br>Linguistic Inquiry and Word Count [65]
**Stigmatizing - Destigmatizing**	Theory of linguistic discrimination [51]
**Personal - Factual**	Coh-Metrix [62]<br>SentiWordNet [67,68]

References

1. Voloshinov VN, Bachtin MM. Marxism and the Philosophy of Language. Harvard University Press; 1986. Available from: https://play.google.com/store/books/details?id=fIPuRyFvDKIC ISBN:9780674550988

2. White PRR. Taking Bakhtin Seriously: Dialogic Effects in Written, Mass Communicative Discourse. University of South Wales 2010;

3. Martin J, White PRR. The Language of Evaluation: Appraisal in English. Springer; 2007. Available from: https://play.google.com/store/books/details?id=I-J8DAAAQBAJ ISBN:9780230511910

4. Alpert JM, Morris BB, Thomson MD, Matin K, Sabo RT, Brown RF. Patient access to clinical notes in oncology: A mixed method analysis of oncologists' attitudes and linguistic characteristics towards notes. Patient Educ Couns 2019 Oct 1;102(10):1917–1924. doi: 10.1016/j.pec.2019.05.008

5. Blease C, Torous J, Hägglund M. Does Patient Access to Clinical Notes Change Documentation? Front Public Health 2020 Nov 27;8:577896. PMID:33330320

6. Pisciotta M, Denneson LM, Williams HB, Woods S, Tuepker A, Dobscha SK. Providing mental health care in the context of online mental health notes: advice from patients and mental health clinicians. J Ment Health 2019 Feb;28(1):64–70. PMID:30468100

7. Harris E, Rob P, Underwood J, Knapp P, Astin F. Should patients still be copied into their letters? A rapid review. Patient Educ Couns 2018 Dec 1;101(12):2065–2082. doi: 10.1016/j.pec.2018.06.014

8. Lobb EA, Butow PN, Kenny DT, Tattersall MH. Communicating prognosis in early breast cancer: do women understand the language used? Med J Aust 1999 Sep 20;171(6):290–294. PMID:10560442

9. Falkenstein A, Tran B, Ludi D, Molkara A, Nguyen H, Tabuenca A, Sweeny K. Characteristics and Correlates of Word Use in Physician-Patient Communication. Ann Behav Med 2016 Oct;50(5):664–677. PMID:26968166

10. Wibe T, Ekstedt M, Hellesø R, Øyri K, Slaughter L. Clinical documentation as a source of information for patients–possibilities and limitations. Medinfo IOS Press; 2013;793–797. doi: 10.3233/978-1-61499-289-9-793

11. Bock B. "Leichte Sprache": Abgrenzung, Beschreibung und Problemstellungen aus Sicht der Linguistik. In: Jekat SJ, Jüngst HE, Schubert K, Villinger C, editors. Sprache barrierefrei gestalten Perspektiven aus der Angewandten Linguistik Berlin: Frank & Timme; 2014. p. 17–51.

12. Kellermann G. Leichte und Einfache Sprache – Versuch einer Definition. Polit Zeitgesch 2014;64(9 – 11):7 – 10.

13. Langer I, von Thun FS, Tausch R, Höder J. Sich verständlich ausdrücken. s3.eu-central-1.amazonaws.com; 1999. Available from: https://s3.eu-central-1.amazonaws.com/coverpubl-erv-01/54376/SP/9783497025329_inhaltsverzeichnis.pdf [accessed Nov 5, 2022]

14. Alpert JM, Krist AH, Aycock RA, Kreps GL. Applying Multiple Methods to Comprehensively Evaluate a Patient Portal's Effectiveness to Convey Information to Patients. J Med Internet Res 2016 May 17;18(5):e112. PMID:27188953

15. Choi HH, Kotsenas AL, Chen JV, Bronsky C, Roth CJ, Kohli MD. Multi-institutional Experience with Patient Image Access Through Electronic Health Record Patient Portals. J Digit Imaging 2022 Apr;35(2):320–326. PMID:35022926

16. Crucefix AL, Fleming APL, Lebus CS, Slowther A-M, Fritz Z. Sharing a written medical summary with patients on the post-admission ward round: A qualitative study of clinician and patient experience. J Eval Clin Pract Wiley; 2021 Dec;27(6):1235–1242. PMID:33960593

17. Erlingsdóttir G, Petersson L, Jonnergård K. A Theoretical Twist on the Transparency of Open Notes: Qualitative Analysis of Health Care Professionals' Free-Text Answers. J Med Internet Res jmir.org; 2019 Sep 25;21(9):e14347. PMID:31573905

18. Grünloh C, Cajander Å, Myreteg G. "The record is our work tool!"—Physicians' framing of a patient portal in Sweden. J Med Internet Res JMIR Publications Inc.; 2016 Jun 27;18(6):e167. doi: 10.2196/jmir.5705

19. Keuper J, Batenburg R, van Tuyl L, Verheij R. General Practices' Experiences With Patients' Web-Based Access to Medical Records: Survey Study. J Med Internet Res 2023 Apr 7;25:e41832. PMID:37027195

20. Leonard SM, Zackula R, Wilcher J. Attitudes and Experiences of Clinicians After Mandated Implementation of Open Notes by the 21st Century Cures Act: Survey Study. J Med Internet Res 2023 Feb 28;25:e42021. PMID:36853747

21. Muli I, Scandurra I, Cajander Å, Hägglund M. Healthcare Professionals' Experiences of the Work Environment After Patients' Access to Their Electronic Health Records - A Qualitative Study in Primary Care. Stud Health Technol Inform 2022 May 25;294:530–534. PMID:35612136

22. Rogers C. The therapeutic relationship: Recent theory and research. Aust J Psychol 1965;17(2):95–108.

23. Rogers CR. Meine Beschreibung einer personenzentrierten Haltung. Zeitschrift für personenzentrierte Psychologie und Psychotherapie 1982;1(1):75–77.

24. Rogers CR. The necessary and sufficient conditions of therapeutic personality changes. Psychotherapy: Therory, Research, Practice, Training 2007;44(3):240–248.

25. Rogers CR. Therapeut und Klient. Grundlagen der Gesprächspsychotherapie. Frankfurt am Main: Fischer; 2013.

26. Gordon S. Appreciative inquiry coaching. International Coaching Psychology Review British Psychological Society; 2008;3(1):17–29. Available from: https://organisationalpsychology.nz/wp-content/uploads/2019/07/International_Coaching_Psychology_Review_Volume_3_No_1_March_2008.pdf#page=21

27. Sidhu SM. Relationship between medical professionals and patients: Exploring the role of nonviolent communication in enhancing engagement. Int J Peace Educ Dev New Delhi Publishers; 2020 Jun 25;8(1). doi: 10.30954/2454-9525.01.2020.6

28. Rosenberg MB. Gewaltfreie Kommunikation: eine Sprache des Lebens. Junfermann

Verlag GmbH; 2007. Available from: https://play.google.com/store/books/details?id=I2Sm9gsxcbkC ISBN:9783873874541

29. Dillo W. Beziehung zum Patienten und seinen Bezugspersonen--ein systemischer Ansatz. Aktion Psychisch Kranke, Peter Weiß, Andreas Heinz (Hg) Qualität therapeutischer Beziehung 1 Auflage ISBN 978-3-88414-641-5 2015;116. Available from: https://www.apk-ev.de/fileadmin/downloads/APK_-_Tagungsberichte_-_Band_041.pdf#page=116

30. Mahler L, Jarchov-Jádi I, Montag C, Gallinat J. Das Weddinger Modell: Resilienz- und Ressourcenorientierung im klinischen Kontext. Psychiatrie Verlag, Imprint BALANCE buch + medien verlag; 2013. Available from: https://play.google.com/store/books/details?id=AjcxEAAAQBAJ ISBN:9783884147917

31. Gassmann D, Grawe K. General change mechanisms: the relation between problem activation and resource activation in successful and unsuccessful therapeutic interactions. Clin Psychol Psychother Wiley; 2006 Jan;13(1):1–11. doi: 10.1002/cpp.442

32. Grawe K, Grawe-Gerber M. Ressourcenaktivierung. Psychotherapeut 1999 Mar 1;44(2):63–73. doi: 10.1007/s002780050149

33. Grawe K. Neuropsychotherapie. Hogrefe Verlag; 2004. Available from: https://play.google.com/store/books/details?id=J2C1caWD4XsC ISBN:9783840918049

34. Wampold BE. The research evidence for the common factors models: A historically situated perspective. The heart and soul of change: Delivering what works in therapy (2nd ed) Washington: American Psychological Association; 2010. p. 49–81. doi: 10.1037/12075-002ISBN:9781433807091

35. Amering M, Schmolke M. Recovery. Das Ende der Unheilbarkeit (5., überarb. Aufl.). Bonn: Psychiatrieverlag.

36. Finzen A. Psychose und Stigma: Stigmabewältigung - zum Umgang mit Vorurteilen und Schuldzuweisungen. Bonn: Psychiatrie-Verlag; 2001. Available from: https://play.google.com/store/books/details?id=Lz9cSQAACAAJ ISBN:9783884142547

37. Finzen A. Schizophrenie, die Krankheit verstehen. Bonn: Psychiatrie Verlag; 2004.

38. Sonnenschein AR, Hofmann SG, Ziegelmayer T, Lutz W. Linguistic analysis of patients with mood and anxiety disorders during cognitive behavioral therapy. Cogn Behav Ther 2018 Jul;47(4):315–327. PMID:29345528

39. Mazor KM, Beard RL, Alexander GL, Arora NK, Firneno C, Gaglio B, Greene SM, Lemay CA, Robinson BE, Roblin DW, Walsh K, Street RL Jr, Gallagher TH. Patients' and family members' views on patient-centered communication during cancer care. Psychooncology 2013 Nov;22(11):2487–2495. PMID:23780672

40. Moth EB, Parry J, Stockler MR, Beale P, Blinman P, Della-Fiorentina S, Kiely BE. Doctor-to-doctor communication of prognosis in metastatic cancer: a review of letters from medical oncologists to referring doctors. Intern Med J Wiley; 2015 Sep;45(9):909–915. PMID:25851689

41. Harris RB, Paradice D. An investigation of the computer-mediated communication of emotions. J Appl Sci Res 2007;3(12):2081–2090. Available from: https://www.academia.edu/download/51530862/An_Investigation_of_the_Computer-me

diate20170127-4600-10jjoc8.pdf

42. Shaver P, Schwartz J, Kirson D, O'Connor C. Emotion knowledge: further exploration of a prototype approach. J Pers Soc Psychol 1987 Jun;52(6):1061–1086. PMID:3598857

43. Salzer MS. Consumer empowerment in mental health organizations: concept, benefits, and impediments. Adm Policy Ment Health 1997 May;24(5):425–434. PMID:9239946

44. Swift C, Levin G. Empowerment: An emerging mental health technology. J Prim Prev 1987 Sep;8(1-2):71–94. PMID:24272076

45. Alegría M, Polo A, Gao S, Santana L, Rothstein D, Jimenez A, Hunter ML, Mendieta F, Oddo V, Normand S-L. Evaluation of a patient activation and empowerment intervention in mental health care. Med Care 2008 Mar;46(3):247–256. PMID:18388839

46. Staples LH. Powerful Ideas About Empowerment. Adm Soc Work Routledge; 1990 Sep 20;14(2):29–42. doi: 10.1300/J147v14n02_03

47. Linhorst DM, Eckert A. Conditions for Empowering People with Severe Mental Illness. Soc Serv Rev The University of Chicago Press; 2003 Jun 1;77(2):279–305. doi: 10.1086/373909

48. Chamberlin J, Schene A. A working definition of empowerment ▼. Psychiatr Rehabil J 1997;20:43–46. Available from: http://polkcountypbsn.org/wp-content/uploads/2012/10/Working-definition-of-empowerment.pdf [accessed Nov 6, 2022]

49. Himmelstein G, Bates D, Zhou L. Examination of Stigmatizing Language in the Electronic Health Record. JAMA Netw Open jamanetwork.com; 2022 Jan 4;5(1):e2144967. PMID:35084481

50. Weiner SG, Lo Y-C, Carroll AD, Zhou L, Ngo A, Hathaway DB, Rodriguez CP, Wakeman SE. The incidence and disparities in use of stigmatizing language in clinical notes for patients with substance use disorder. J Addict Med Ovid Technologies (Wolters Kluwer Health); 2023 Jan 31; PMID:36719760

51. Drożdżowicz A, Peled Y. The complexities of linguistic discrimination. Philos Psychol Informa UK Limited; 2024 Mar 9;1–24. doi: 10.1080/09515089.2024.2307993

52. Alpert JM, Morris BB, Thomson MD, Matin K, Geyer CE, Brown RF. OpenNotes in oncology: oncologists' perceptions and a baseline of the content and style of their clinician notes. Transl Behav Med academic.oup.com; 2019 Mar 1;9(2):347–356. PMID:29596633

53. Blease C, Torous J, Dong Z, Davidge G, DesRoches C, Kharko A, Turner A, Jones R, Hägglund M, McMillan B. Patient Online Record Access in English Primary Care: Qualitative Survey Study of General Practitioners' Views. J Med Internet Res 2023 Feb 22;25:e43496. PMID:36811939

54. Chimowitz H, O'Neill S, Leveille S, Welch K, Walker J. Sharing Psychotherapy Notes with Patients: Therapists' Attitudes and Experiences. Soc Work academic.oup.com; 2020 Apr 1;65(2):159–168. PMID:32236447

55. Johansen MA, Kummervold PE, Sørensen T, Zanaboni P. Health Professionals' Experience with Patients Accessing Their Electronic Health Records: Results from an Online Survey. Stud Health Technol Inform ebooks.iospress.nl; 2019 Aug

21;264:504–508. PMID:31437974

56. Zanaboni P, Kristiansen E, Lintvedt O, Wynn R, Johansen MA, Sørensen T, Fagerlund AJ. Impact on patient-provider relationship and documentation practices when mental health patients access their electronic health records online: a qualitative study among health professionals in an outpatient setting. BMC Psychiatry 2022 Jul 28;22(1):508. PMID:35902841

57. Zellmer BM, Nacht CL, Coller RJ, Hoonakker PLT, Smith CA, Sklansky DJ, Dean SM, Smith W, Sprackling CM, Ehlenfeldt BD, Kelly MM. BedsideNotes: Sharing Physicians' Notes With Parents During Hospitalization. Hosp Pediatr publications.aap.org; 2021 May;11(5):503–508. PMID:33795371

58. Walker J, Leveille SG, Ngo L, Vodicka E, Darer JD, Dhanireddy S, Elmore JG, Feldman HJ, Lichtenfeld MJ, Oster N, Ralston JD, Ross SE, Delbanco T. Inviting patients to read their doctors' notes: patients and doctors look ahead: patient and physician surveys. Ann Intern Med acpjournals.org; 2011 Dec 20;155(12):811–819. PMID:22184688

59. Denneson LM, Cromer R, Williams HB, Pisciotta M, Dobscha SK. A Qualitative Analysis of How Online Access to Mental Health Notes Is Changing Clinician Perceptions of Power and the Therapeutic Relationship. J Med Internet Res jmir.org; 2017 Jun 14;19(6):e208. PMID:28615152

60. Drinkwater J, Stanley N, Szilassy E, Larkins C, Hester M, Feder G. Juggling confidentiality and safety: a qualitative study of how general practice clinicians document domestic violence in families with children. Br J Gen Pract 2017 Jun;67(659):e437–e444. PMID:28137783

61. King G, Maxwell J, Karmali A, Hagens S, Pinto M, Williams L, Adamson K. Connecting Families to Their Health Record and Care Team: The Use, Utility, and Impact of a Client/Family Health Portal at a Children's Rehabilitation Hospital. J Med Internet Res 2017 Apr 6;19(4):e97. PMID:28385680

62. Graesser AC, McNamara DS, Louwerse MM, Cai Z. Coh-metrix: analysis of text on cohesion and language. Behav Res Methods Instrum Comput Springer Science and Business Media LLC; 2004 May;36(2):193–202. PMID:15354684

63. Flesch R. A new readability yardstick. J Appl Psychol 1948 Jun;32(3):221–233. PMID:18867058

64. White PR. Appraisal theory. The international encyclopedia of language and social interaction prrwhite.info; 2015;3:1–7. Available from: https://www.prrwhite.info/prrwhite,%202015,%20Appraisal%20theory,%20Wiley%20Encylopedia.pdf

65. Pennebaker JW, Booth RJ, Boyd RL, Francis ME. Linguistic Inquiry and Word Count: LIWC2015. Austin, TX: Pennebaker Conglomerates; 2015.

66. Brown P, Levinson S. Politeness: Some Universals in Language Usage. Cambridge: Cambridge University Press; 1987.

67. Baccianella S, Esuli A, Sebastiani F. SentiWordNet 3.0: An enhanced lexical resource for sentiment analysis and opinion mining. LREC lrec-conf.org; 2010 May 1; Available from: http://lrec-conf.org/proceedings/lrec2010/pdf/769_Paper.pdf

68. Esuli A, Sebastiani F. SENTIWORDNET: A publicly available lexical resource for opinion

mining. LREC researchgate.net; 2006;417–422. Available from: https://www.researchgate.net/profile/Jose-Luis-Vigil/post/Need-assistance-in-proposal-for-Masters-Dissertation/attachment/59d64c8879197b80779a64d9/AS%3A484605510852611%401492550365376/download/Miner%C3%ADa+de+Opiniones.+Lingu%C3%ADstica+Computacional.pdf
